# Fatal Eucalyptus Oil Poisoning in an Adult Male: A Case Report With Comprehensive Autopsy and Histopathological Findings

**DOI:** 10.7759/cureus.83053

**Published:** 2025-04-26

**Authors:** Deepika Suresh, Sibi VijayaKumar, Priyadarshee Pradhan, Subalakshmi Balasubramanian

**Affiliations:** 1 Forensic Medicine and Toxicology, Sri Ramachandra Institute of Higher Education and Research, Chennai, IND; 2 Pathology, Sri Ramachandra Institute of Higher Education and Research, Chennai, IND

**Keywords:** acute tubular necrosis, essential oil, eucalyptus oil, eucalyptus poisoning, generalized tonic-clonic seizures

## Abstract

Eucalyptus oil is a widely available essential oil with therapeutic applications, but its toxic effects following ingestion are significant. We report a fatal case of eucalyptus oil poisoning in an adult male who mistakenly ingested 15 mL of eucalyptus oil stored in a cough syrup container. The patient developed generalized tonic-clonic seizures within minutes, followed by unresponsiveness and respiratory depression. Despite intubation and supportive treatment, he suffered a cardiac arrest and was revived after cardiopulmonary resuscitation (CPR). However, multiple seizure episodes persisted, and he was transferred to a tertiary care center, where he succumbed to death. Autopsy findings revealed corrosive damage to the tongue, trachea, stomach, and intestines, along with congestion in multiple organs. Histopathological examination showed extensive damage, including pulmonary emphysema, macrovesicular steatosis, and acute tubular necrosis. This case highlights the multi-organ effects of eucalyptus oil poisoning and underscores the importance of public awareness regarding its potential lethality.

## Introduction

Eucalyptus oil is commonly used for its antimicrobial and decongestant properties. However, its ingestion, particularly in children and adults at high doses, can lead to severe toxic manifestations [1,2]. 1,8-cineole, also known as eucalyptol, is the main toxic component and has been linked to respiratory issues, convulsions, and depression of the central nervous system. Severe poisoning can cause coma, status epilepticus, and aspiration pneumonitis, whereas mild poisoning manifests as nausea, vomiting, dizziness, and ataxia [3,4].

While most reports indicate that a typical lethal dose in adults is between 30 and 45 mL, individual susceptibility varies significantly based on factors, including pre-existing conditions and metabolic differences [5]. Symptoms usually manifest within 10 to 30 minutes and include convulsions, respiratory distress, and central nervous system depression [6]. Aspiration can result in deadly pneumonitis and intensify the toxic consequences [7]. Airway protection, seizure control, and circulatory stabilization remain the main supportive measures used to treat eucalyptus oil toxicity [7,8].

While eucalyptus oil poisoning has been well-documented in pediatric populations, comprehensive reports of fatal adult cases with detailed autopsy and histopathological findings are scarce in the literature [9,10]. This case report addresses this gap by providing an in-depth analysis of the systemic involvement observed in a fatal adult case. The correlation between clinical manifestations and specific tissue damage patterns offers valuable insights into the pathophysiological mechanisms of eucalyptus oil toxicity. Understanding these mechanisms is crucial for developing more targeted therapeutic approaches and improving outcomes in severe cases [11]. This report discusses the clinical course, autopsy findings, and histopathological evidence of multi-organ toxicity in a fatal case of eucalyptus oil poisoning.

## Case presentation

Clinical history

A 36-year-old male with a one-week history of low-grade intermittent fever, cough, and cold was under symptomatic treatment with prescribed antitussives. On 12/09/2024, at approximately 06:50 PM, he accidentally consumed approximately 15 mL of eucalyptus oil, mistaking it for cough syrup. Within 10 minutes of ingestion, he developed generalized tonic-clonic seizures (GTCS) lasting around 10 minutes. A second GTCS episode occurred en route to the hospital.

At the outside hospital, he was found to be unresponsive (Glasgow Coma Scale (GCS) E1V1M1), with no peripheral pulses and cardiac arrest. He was immediately intubated (ET tube size 7.5, fixed at 26 cm), placed on bag-valve-mask ventilation, and resuscitation was initiated. Return of spontaneous circulation (ROSC) was achieved after 20 minutes of cardiopulmonary resuscitation (CPR). Arterial blood gas analysis (ABG) at this point revealed severe metabolic acidosis (pH 7.086, pCO₂ 58.7, pO₂ 27, HCO₃ 6.6, lactate 8.72), and supportive therapy was initiated. He was referred to the tertiary center for advanced care.

On arrival at the tertiary center (13/09/2024, 12:10 AM), the patient was sedated and intubated (ET size 8.0, fixed at 21 cm). Vitals were HR 120/min, BP not recordable, and peripheral capillary oxygen saturation (SPO₂) 98%. Ryles tube drainage showed 250 mL of blood mixed with food particles. He was managed with inotropes (noradrenaline, adrenaline, dopamine), corticosteroids, and fluid therapy. Serial ABGs showed persistent metabolic acidosis despite intervention.

At 02:39 AM, the patient suddenly developed asystole. CPR was initiated as per the advanced cardiovascular life support (ACLS) protocol. After two doses of adrenaline and four cycles of CPR, ROSC was achieved again. However, the patient again progressed to bradycardia and persistent asystole at 02:59 AM. Despite 20 minutes of advanced cardiac life support, the patient could not be revived. He was declared dead at 03:25 AM on 13/09/2024, and his body was sent for postmortem examination.

External examination and gross findings

**Figure 1:**
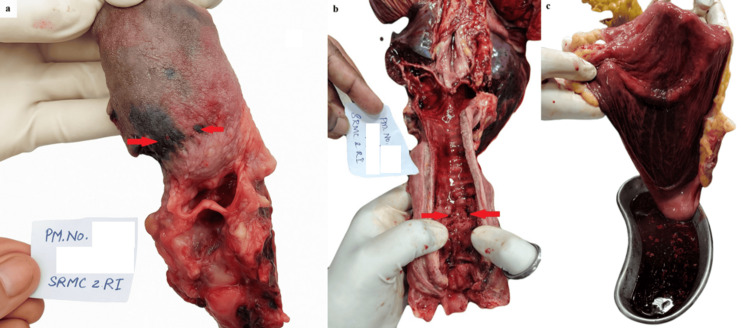
Autopsy findings showing corrosive effects (1a) Blackish discoloration and corrosion of the tongue (red arrows); (1b) Hemorrhagic corrosive spots in the trachea (red arrows); (1c) Hemorrhagic corrosion of the gastric mucosa

The autopsy revealed blackish discoloration with evidence of corrosion on the tongue (Figure 1a). The trachea displayed hemorrhagic corrosive spots (Figure 1b). Multiple petechial hemorrhages are present over the entire surface of both lungs. The gastrointestinal tract showed hemorrhagic corrosion affecting both the mucosa of the stomach (Figure 1c) and the intestines. The stomach contents included an oily, shining fluid with a strong, characteristic woody odor, highly suggestive of eucalyptus oil. The kidney appeared congested and hemorrhagic, while the liver exhibited a nutmeg appearance with hemorrhagic changes. Notably, all organs demonstrated marked congestion.

Toxicological analysis

Chemical analysis of the viscera was performed as part of the comprehensive autopsy examination. Toxicological screening confirmed the presence of eucalyptus oil components in the visceral samples, corroborating the circumstantial evidence of accidental ingestion. This toxicological confirmation, alongside the characteristic odor of the stomach contents and the reported clinical history, provided conclusive evidence of eucalyptus oil poisoning as the cause of death.

Histopathological examination results

**Figure 2:**
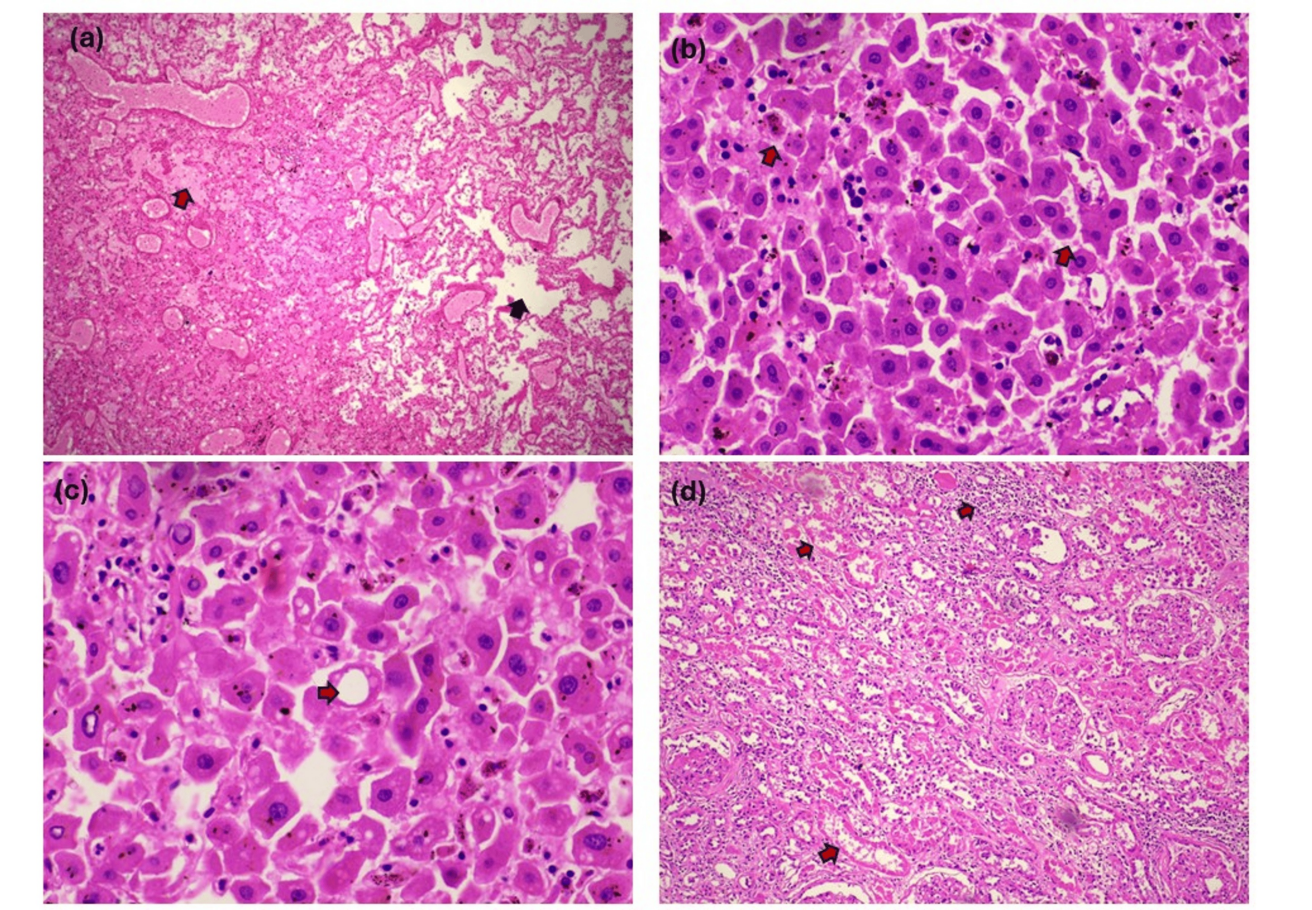
Histopathology findings (2a) Lung parenchyma showing alveolar dilatation(black arrow) and pulmonary edema (red arrow) (H&E, 100x); (2b) Hepatic sinusoidal congestion (red arrows) (H&E, 400x); (2c) Liver parenchyma showing macrovesicular steatosis (red arrow) (H&E, 400x); (2d) Kidney section showing acute tubular necrosis (red arrows) (H&E, 100x)

Microscopic analysis of the lungs revealed lung parenchyma with dilatation of alveoli and features of pulmonary edema (Figure 2a). The liver showed sinusoidal congestion (Figure 2b) and macrovesicular steatosis (Figure 2c), while the kidneys displayed acute tubular necrosis (Figure 2d). In contrast, both cardiac muscle and brain tissue showed no significant pathological changes upon histopathological examination.

## Discussion

This article highlights the multi-organ harm that eucalyptus oil ingestion causes, including renal dysfunction, hepatic injury, status epilepticus, and central nervous system depression. Our patient's neurological symptoms started quickly, which is consistent with other case reports that show seizures within 10 to 15 minutes of consumption [6]. Unlike pediatric cases, where recovery is common within 24 hours, adult cases with severe exposure may lead to fatal outcomes when complicated by underlying conditions or delayed treatment [9].

Severe eucalyptus oil poisoning can lead to metabolic acidosis, a condition often seen in patients with prolonged seizures and multi-organ dysfunction [3]. Compared to the two cases described by Kumar et al., who presented with mild tachycardia, leukocytosis, normal renal function, and mild elevations in liver enzymes, our patient demonstrated a far more fulminant course. While metabolic acidosis was a common finding (pH 6.983 and 7.042 in their cases vs. 7.086 in ours), our patient exhibited a significantly elevated lactate (8.72 mmol/L) and profound hemodynamic collapse necessitating multiple inotropes. Unlike their patients who remained hemodynamically stable, our case progressed to repeated seizures, two episodes of cardiac arrest, and ultimately refractory asystole, highlighting a more severe toxic response despite a comparable volume of eucalyptus oil ingestion (~15 mL) [12].

The severe hemorrhagic erosions observed in the stomach and intestines of our patient are consistent with the direct corrosive effects of eucalyptus oil on mucosal surfaces. Eucalyptus oil contains multiple terpenoids, particularly 1,8-cineole, which can cause direct cellular damage to epithelial tissues [13]. The pathophysiology of eucalyptus oil-induced GI erosions involves multiple mechanisms. First, the components of eucalyptus oil are lipophilic, which enables quick entry into the membranes of epithelial cells and disrupts their structural integrity [14]. Second, eucalyptus oil can trigger inflammatory cascades, leading to increased vascular permeability and mucosal hemorrhage, as observed in our patient [15]. Third, the oil's ability to denature proteins contributes to the corrosive effects seen throughout the GI tract [16]. A study by Santos and Rao demonstrated that 1,8-cineole affects membrane permeability in gastrointestinal epithelial cells, potentially explaining the corrosive effects observed in our case [13]. Previous research by Darben et al. also reported that eucalyptus oil can cause erosive gastritis even at lower doses than were ingested in our case [11].

In addition, since eucalyptus oil is extremely volatile and can directly injure the lungs when inhaled, aspiration pneumonitis is a major risk factor [10]. Because of its high lipid solubility, eucalyptus oil is widely distributed throughout the body, which increases toxicity and slows elimination [17]. The histopathological findings in our case reveal important insights into the multisystem toxicity of eucalyptus oil. The pulmonary findings of alveolar dilatation and pulmonary edema likely result from a combination of direct toxic effects and secondary complications of seizures and respiratory depression. The presentation of pulmonary edema is consistent with findings by Tibballs, who observed significant respiratory compromise in cases of essential oil toxicity [4]. However, the other expected classical findings of diffuse alveolar damage, chemical pneumonitis, or acute respiratory distress syndrome, as documented by Webb and Pitts in their study [10], were not observed by us. The liver showed macrovesicular steatosis and sinusoidal congestion, indicating hepatocellular injury. The findings are consistent with studies by Fromenty and Pessayre, who showed that steatosis can result from specific plant toxins interfering with hepatic lipid metabolism [18]. The pattern of liver damage points to oxidative stress and mitochondrial dysfunction, which are frequently linked to exposure to toxins [19].

As we observed in our case, severe episodes of essential oil toxicity have been known to cause renal tubular necrosis. The mechanism likely involves direct toxic effects on tubular epithelial cells, combined with hypoperfusion due to cardiovascular compromise during the patient's cardiac arrest episodes [17]. Similar patterns of renal injury have been observed in other cases of plant toxin ingestion, as reported by Gurr and Scroggie [8].

The absence of significant pathological changes in cardiac and brain tissue despite the patient's clinical presentation of seizures and cardiac arrest is noteworthy. This suggests that the primary mechanism of cardiorespiratory collapse may have been related to severe metabolic derangements, hypoxia from pulmonary dysfunction, or neuronal functional impairment rather than structural damage. Similar findings were reported by Patel and Wiggins in their case series, where functional neurological symptoms predominated despite minimal structural changes on post-mortem examination [6].

Several studies indicate that there is no direct correlation between the amount of eucalyptus oil ingested and symptom severity, making early supportive management critical. The absence of a specific antidote further complicates treatment [20]. Our case illustrates the potentially lethal consequences of eucalyptus oil in adult patients with individual susceptibility factors, as the patient passed away from its toxic effects despite immediate medical assistance, including intubation and cardiac resuscitation. This case highlights that while typical lethal doses are reported to be 30-45 mL, severe idiosyncratic reactions or complications can occur at lower volumes in susceptible individuals [5,20].

## Conclusions

Eucalyptus oil ingestion can lead to rapidly fatal poisoning due to neurotoxicity, multi-organ dysfunction, and cardiac failure. The corrosive effects on mucosal surfaces, combined with CNS depression and aspiration risk, make early airway management and seizure control crucial in treatment. To avoid unintentional poisoning, the public must be made aware of the risks associated with consuming eucalyptus oil. The unique contribution of this case report lies in its comprehensive documentation of histopathological findings in a fatal adult case, providing valuable insights into the pathophysiological mechanisms of eucalyptus oil toxicity. Further research is needed to develop specific treatment protocols for severe eucalyptus oil poisoning to improve outcomes in these challenging cases.
